# Long non-coding RNA LINC01116 is activated by EGR1 and facilitates lung adenocarcinoma oncogenicity via targeting miR-744-5p/CDCA4 axis

**DOI:** 10.1186/s12935-021-01994-w

**Published:** 2021-06-05

**Authors:** Ping Ren, Liang Chang, Xiaodong Hong, Lei Xing, Hong Zhang

**Affiliations:** Department of Thoracic Surgery, The First Hospital of Jilin University, No. 71, Xinmin Road, Changchun, 130021 Jilin China

**Keywords:** Lung adenocarcinoma, LINC01116, EGR1, MiR-744-5p, CDCA4, Malignant behaviors

## Abstract

**Background:**

Lung adenocarcinoma (LAD) is one of the most frequently diagnosed pathological categories of human lung cancer. Nevertheless, the link between long non-coding RNA (lncRNA) LINC01116 and LAD remains poorly investigated.

**Methods:**

QRT-PCR and western blot were applied for quantifying the expression of RNAs and proteins. Both functional experiments assays in vitro and xenografts model in vivo were implemented for analyzing LINC01116 function in LAD while molecular relationship among RNAs was investigated via mechanism experiments.

**Results:**

LINC01116 was expressed at an abnormally high level in LAD, which was induced by transcription activator EGR1. LINC01116 depletion restrained proliferation, migration and invasion, yet facilitated apoptosis of LAD cells. MiR-744-5p could bind to LINC01116. MiR-744-5p inhibitor reversed the inhibitory effects of silencing LINC01116 on LAD malignant behaviors. In addition, cell division cycle-associated protein 4 (CDCA4) shared binding sites with miR-744-5p. Silencing LINC01116 elicited decline in CDCA4 mRNA and protein levels. Moreover, CDCA4 up-regulation could counteract the biological effects of LINC01116 knockdown on LAD cells.

**Conclusion:**

Our data revealed that LINC01116 promoted malignant behaviors of LAD cells by targeting miR-744-5p/CDCA4 axis, implying the theoretical potential of LINC01116 as a novel target for LAD treatment.

**Supplementary Information:**

The online version contains supplementary material available at 10.1186/s12935-021-01994-w.

## Background

With increasing morbidity, lung cancer is a leading cause of cancer-associated deaths worldwide, posing a threat to the health and life of patients [1]. Adenocarcinoma is a predominant pathological form of lung cancer [2]. Over the past decade, despite substantial progress has been made in therapeutic strategies, including chemotherapy, radiotherapy, surgery and targeting therapies, the overall prognosis for lung adenocarcinoma (LAD) remains far from satisfying. Notably, the 5-year survival rate jumped to around 21% combined with all different clinical stages, which partly was attributed to the aggressive characteristics of LAD in the clinical course [3, 4]. LAD could induce metastasis in contralateral lung, lymph nodes, and sometimes in distant organs, such as bones and brain [5]. Efforts in exploring the unidentified biomarkers that are associated with the aggressiveness of LAD are relatively valuable for discovering novel promising therapeutic targets.

Consisting of more than 200 nucleotides, long non-coding RNA (lncRNA) is a subtype of non-coding RNAs without protein-coding ability [6]. A large amount of lncRNAs have been uncovered to exert pivotal influences on progression of multiple malignancies [7]. The close association between the abnormal expression level of lncRNA and tumor cell growth and migration has been fathomed out in the past publications [8, 9]. Moreover, it has been suggested in accumulating literatures that lncRNAs could exert specific functions, with competing endogenous RNAs (ceRNAs) network involved, by serving as miRNA sponges in post-transcriptional mediation [10]. LINC01116 was initially reported to bind to target genes and exerted oncogenic effects on prostate cancer [11]. It has been proved that LINC01116 could promote gefitinib resistance in non-small cell lung cancer via modulation of IFI44 [12]. However, its performance and potential mechanism in LAD remain unclear.

MiRNAs are capable of repressing the expression of specific downstream target post-transcriptionally. By regulating the expression level of target genes, miRNAs played a crucial part in cellular activities, including cell growth, invasion, and apoptosis [13]. Extensive studies revealed the dysregulation of miRNAs in different tumor types had profound implication in carcinogenesis process [14]. MiR-744-5p has been discovered to possess tumor suppressing property and positively related with apoptosis signaling in ovarian cancer [15]. LINC01116 was depicted to exacerbate malignant behaviors of lung squamous cell carcinoma cells via regulation of miR-744-5p/SCN1B axis [16]. However, the interaction between miR-744-5p and LINC01116 has never been uncovered in LAD.

Cell division cycle-associated protein 4 (CDCA4) has been reported to interact with miRNA-15a and contribute to cells growth and invasion of malignant melanoma in vitro [17]. However, the underlying relationship between miR-744-5p and CDCA4 in LAD has never been proved. In this study, the main aim was to explore the biological functions of LINC01116 and its potential regulatory mechanism in the malignant behaviors of LAD.

## Materials and methods

### Cell lines and culture

Human LAD cell lines (H1975, PC9, A549, SPCA-1) and normal human lung epithelial cell line (HBE), all from the ATCC (Manassas, VA), were cultured with 5% CO_2_ at 37 °C in the DMEM medium (Invitrogen, Carlsbad, CA). 10% fetal bovine serum (FBS; Gibco, Waltham, MA) and 1% penicillin–streptomycin solution (Invitrogen) were served as supplements for the medium.

### Quantitative real-time PCR (qRT-PCR)

The extract of total RNA was obtained with the help of TRIzol Regent (Invitrogen) under manufacturer’s instruction. Subsequent to reverse transcription, cDNA was used for qPCR on Step-One Plus Real-Time PCR System (Applied Biosystems, Foster City, CA) using SYBR^®^ Premix Ex Taq™ II (Takara, Shiga, Japan). Gene expression was standardized to GAPDH or U6, calculated with 2^−ΔΔCt^ method.

### Plasmid transfection

The LINC01116-specifc shRNAs and control shRNAs were procured from Genepharma Company (Shanghai, China) and transfected into A549 and PC9 cell samples for 48 h by Lipofectamine 2000 (Invitrogen). In addition, pcDNA3.1/EGR1, pcDNA3.1/LINC01116, pcDNA3.1/CDCA4, negative control pcDNA3.1, as well as the miR-744-5p mimics/inhibitor and NC mimics/inhibitor were all designed by Genepharma Company.

### EdU staining

Transfected LAD cells were seeded into the 96-well plates, followed with the addition of 100 μL of EdU. After being cultivated for 3 h, the cells were subject to fixation with 4% paraformaldehyde and permeabilized in 0.5% Troxin X-100. Subsequent to nuclear staining with DAPI in the dark, the observation of proliferative cells was conducted by means of fluorescent microscope (Leica, Wetzlar, Germany) and calculated with the help of Image J software.

### Colony formation

Cell samples of A549 and PC9 were cultured in the 6-well plates with a density of 500 cells per well for 14 days. Samples were afterwards subject to fixation with 4% paraformaldehyde and staining with 0.5% crystal violet. The colony number was counted manually.

### Flow cytometer for apoptosis

Transfected A549 and PC9 cell samples were cultivated in 6-well plates for 48 h. Cells were washed by PBS thrice. Samples were dyed in Annexin V-FITC/PI detection kit (Invitrogen) at 4 °C for 15 min. Flow cytometer (BD Biosciences, Franklin Lakes, NJ) was applied for detection of cell apoptosis and FlowJo_V10 was applied for calculation of apoptotic cell number.

### Western blot

Total protein was prepared in RIPA lysis buffer (Beyotime, Nantong, China) on ice, then extracted by 12% SDS-PAGE and shifted to PVDF membranes (Millipore, Billerica, MA). Membranes were sealed for 1 h with 5% skim milk and incubated with primary antibodies including anti-GAPDH (ab9485, abcam), anti-Bax (ab32503, abcam), anti-Bcl-2 (ab32124, abcam), anti-caspase 3 (ab3235, abcam), anti-cleaved caspase 3 (ab2302, abcam), anti-CDCA4 (ab227953, abcam), anti-ki-67 (ab270650, abcam), anti-PCNA (ab29, abcam), and anti-EGR1 (ab194357, abcam) respectively all night at 4 °C. After being washed in TBST, secondary antibodies tagged with HRP were added and incubated for 1 h at 37 °C. The protein bands were quantified by ECL Prime Western Blotting Detection reagent (GE Healthcare, Chicago, IL).

### Transwell assays

Transwell assays for invasion or migration were conducted by application of the transwell membrane (Corning Incorporated, Corning, New York) coated with or without Matrigel (BD Biosciences). Evaluation of invasion or migration ability of A549 and PC9 cell samples was achieved via counting migrated or invaded cells under microscope (10 × 10). 5 randomly selected fields of view in each chamber were analyzed by Image J.

### Chromatin immunoprecipitation (ChIP)

Cell samples were crosslinked in 4% paraformaldehyde at room temperature. Cells were lysed in lysis buffer added with protease and phosphatase inhibitor, and then sonicated to obtain the nuclei part. Subsequently, samples were incubated all night with the protein G beads-bound antibodies against EGR1 or normal control IgG (ab133470, abcam). After reverse crosslinking, the DNA–protein mixture was purified for obtaining DNA, followed by qRT-PCR analysis.

### Luciferase reporter assays

The wild-type (WT) or mutated (Mut) binding sites of EGR1 in LINC01116 promoter were designed and sub-cloned into pGL3 luciferase vector (Promega, Madison, WI), then co-transfected with pcDNA3.1/EGR1 or NC pcDNA3.1 to HEK-293 T cells. The WT or Mut binding sites of miR-744-5p in LINC01116 sequence or CDCA4-3′-UTR were sub-cloned into pmirGLO luciferase vector (Promega), and then co-transfected with miR-744-5p mimics or NC mimics in LAD cell samples. 48 h later, dual-luciferase reporter assay system (Promega) was applied to detect the relative luciferase activity as per the protocol.

### Subcellular fractionation

LAD cell samples were rinsed gently in cold PBS, then lysed on ice and centrifuged. The precipitates containing the cell nucleus was separated from supernatant containing the cell cytoplasm. After being purified, expression levels of LINC01116, GAPDH and U6 were monitored by qRT-PCR.

### Fluorescence in situ hybridization (FISH)

The RNA FISH probe synthesized for LINC01116 was procured from Ribobio (Guangzhou, China) and employed under supplier’s instruction. Cells were fixed with 4% paraformaldehyde for about 10 min and washed three times. After that, the probes were put into the cell culture medium and hybridized for a whole night. Nucleus staining was performed with Hoechst solution. Stained cells were analyzed with fluorescent microscope.

### RNA pull down

The wild-type or mutated miRNAs binding sites in LINC01116 sequence were synthesized and biotin-labeled to acquire Bio-LINC01116-WT/Mut probes. The wild-type or mutated LINC01116 binding sites in miR-744-5p sequence were also synthesized and biotin-labeled to generate the Bio-miR-744-5p-WT/Mut probes. After incubation with cellular protein extracts and beads, qRT-PCR was used to detect the enrichment of RNAs.

### RNA binding protein immunoprecipitation (RIP)

According to the user manual, Magna RNA-binding protein immunoprecipitation kit (Millipore) was applied for RIP assay with antibodies against Ago2 (#2897, Cell signaling) and control IgG. Relative RNA enrichment was assessed by qRT-PCR.

### Xenograft tumor experiment

All animal-associated protocol was approved by the Ethics Committee of Animal Experiments of the First Hospital of Jilin University. 2 × 10^6^ transfected A549 cells were inoculated subcutaneously in the 6-week-old BALB/c-nu mice from Animal Center of the Chinese Academy of Science (Shanghai, China). Tumor volumes were measured every 4 days after being apparently observed and calculated according to the formula: Volume  =  (length  ×  width^2^)/2. Mice were sacrificed after 28 days and the tumors were excised and collected for the measurement of tumor weight with the help of electronic balance.

### Statistical analysis

Experiment data was handled by GraphPad Prism 6.0 software. Student’s t test, one-way analysis of variance (ANOVA) and two-way ANOVA were used in this study. P  <  0.05 was the cutoff value of significance. Continuous variables from three bio-repeats were presented as the mean  ±  SD.

## Results

### LINC01116 is markedly elevated in LAD cells and silencing LINC01116 can restrain the carcinogenic behaviors of LAD cells

**Fig. 1:**
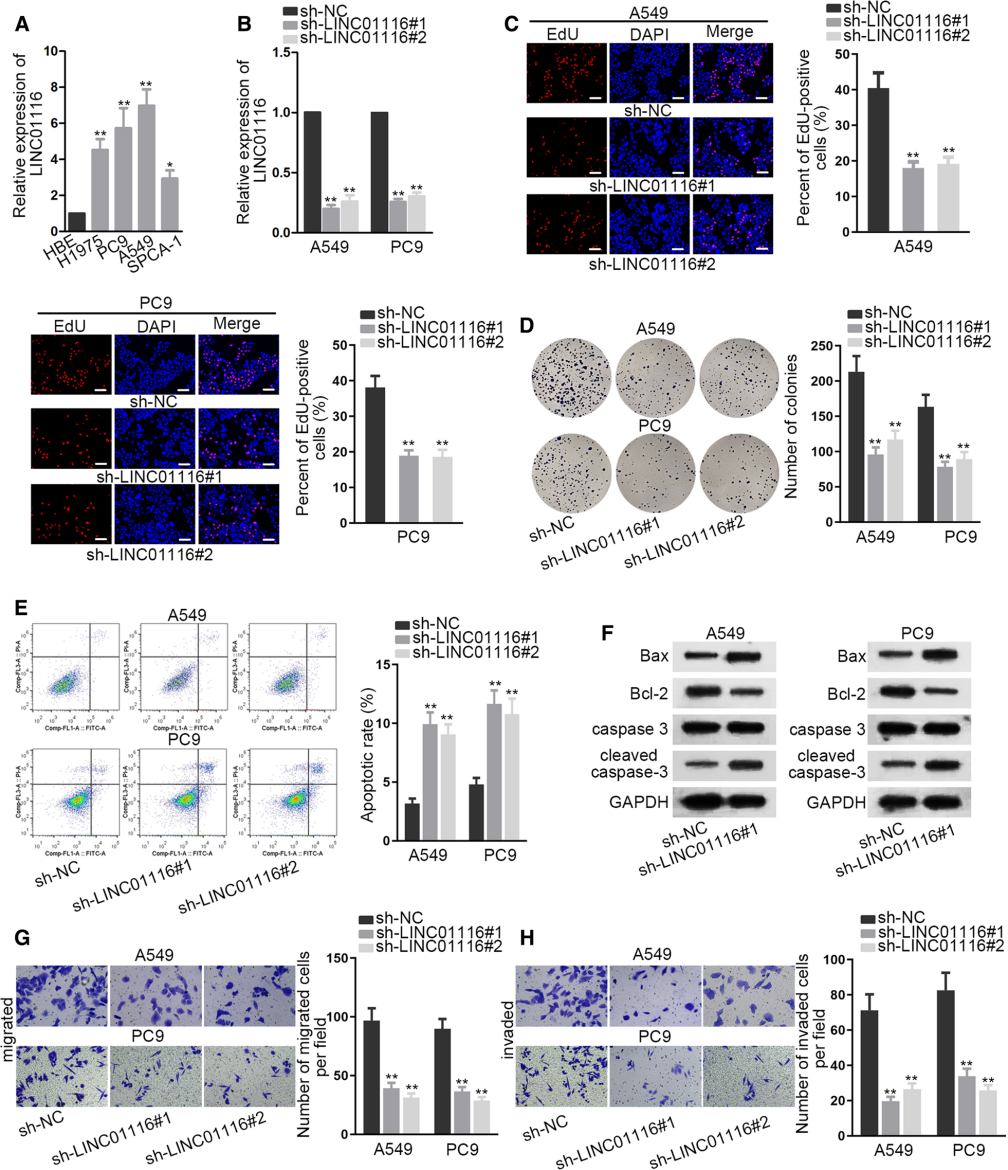
LINC01116 is markedly elevated in LAD cells and silencing LINC01116 can restrain the carcinogenic behaviors of LAD cells. **A** Quantification of LINC01116 expression in different cell lines was conducted via qRT-PCR. **B** The detection of LINC01116 knockdown efficiency was achieved by means of qRT-PCR analysis. **C**, **D** EdU and colony formation assays were employed for assessing cell proliferation capacity after LINC01116 depletion. **E** Apoptosis of A549 and PC9 was evaluated after LINC01116 depletion via flow cytometry. **F** Apoptosis-related proteins were investigated via western blot. **G**, **H **Transwell assays were utilized to evaluate the number of migrated and invaded cells was calculated after LINC01116 depletion by means of transwell assays. One-way ANOVA was applied for analysis. *P  <  0.05, **P  <  0.01

To analyze the potential role of LINC01116 in LAD, qRT-PCR analysis was implemented to detect the expression of LINC01116 in LAD cells. It manifested that LINC01116 was markedly elevated in LAD cells compared with HBE cells (Fig. 1A). We further probed into the function of LINC01116 in LAD. We selected A549 and PC9 cell lines as the objects of further study, since LINC01116 was found most highly expressed in these two cell lines. We successfully silenced the expression of LINC01116 by transfecting sh-LINC01116 in A549 and PC9 cells (Fig. 1B). EdU assay illustrated an attenuation of cell proliferation by silencing LINC01116 (Fig. 1C). The restraining impacts on cell proliferation elicited by LINC01116 silence was further confirmed by colony formation assay (Fig. 1D). Based on the results of western blot, the level of proliferation markers, namely ki-67 and PCNA, decreased after knockdown of LINC01116 (Additional file 1: Figure S1A). Flow cytometry assay showed an increasing apoptosis rate, indicating the promoting impacts of LINC01116 silence on cell apoptosis (Fig. 1E). Western blot showed increased expression of Bax and cleaved caspase-3, reduced expression of Bcl-2, whereas almost unchanged expression of caspase 3 after silencing LINC01116, which validated the outcome of flow cytometry (Fig. 1F). More importantly, transwell assays demonstrated that cell migration and invasion were impeded as a result of silencing LINC01116 (Fig. 1G, H). Hence, it was reasonable to presume that LINC01116 was involved in the advancement of LAD and might play a stimulating role in that process.

### EGR1 activates the transcription of LINC01116

**Fig. 2:**
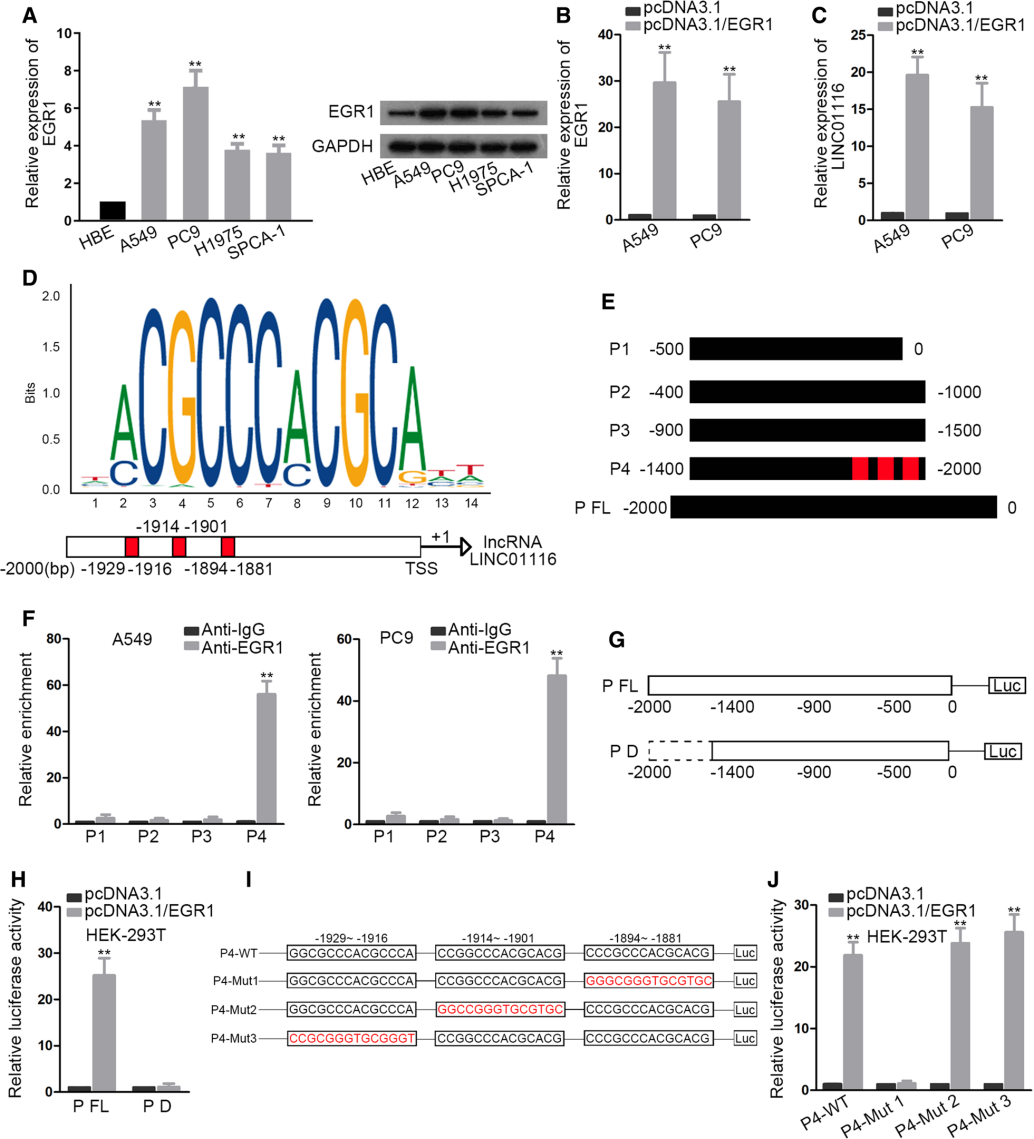
EGR1 activates the transcription of LINC01116. **A** EGR1 expression in LAD cell lines and HBE was investigated by qRT-PCR. One-way ANOVA was applied for analysis. **B** The expression level of EGR1 in A549 and PC9 cells was quantified subsequent to the transfection with pcDNA3.1/EGR1. Student’s t test was applied for analysis. **C** The impacts of EGR1 overexpression on LINC01116 was analyzed by qRT-PCR. Student’s t test was applied for analysis. **D** The DNA binding motif of EGR1 on LINC01116 promoter and three specific binding sites were predicted by JASPAR. **E** LINC01116 promoter was divided into four sectional parts based on predicted binding sites. **F** ChIP assay revealed the interaction of EGR1 with P4 sectional area in LINC01116 promoter region. Student’s t test was applied for analysis. **G** The constructions of full promoter (P FL) and P4 deleted (P D) promoter region were sub-cloned into pGL3 luciferase reporter vector. **H** Luciferase activity was assessed in HEK-293 T cells co-transfected with pcDNA3.1 or pcDNA3.1/EGR1 and pGL3 luciferase reporter vectors carrying P FL or P D region. Student’s t test was applied for analysis. **I** The sequence of P4 wild type and corresponding point mutant sequences, including P4-Mut1, P4-Mut2 and P4-Mut3 were sub-cloned into pGL3 reporter vector. **J** Luciferase reporter assay was conducted for analysis of promoter activity. Student’s t test was applied for analysis. **P  <  0.01

It has been widely reported that transcription factor could activate or repress the transcription activity of lncRNAs, thus impacting the expression level of lncRNAs [18]. By means of UCSC (http://genome.ucsc.edu/) and JASPAR (http://jaspar.genereg.net/), five transcription factor candidates (EGR1, CTCF, YY1, FOXA1, FOXP2) were selected. Only EGR1 knockdown resulted in overt decline in LINC01116 expression (Additional file 1: Figure S1B). Thus, we speculated that EGR1 might be a transcription factor for LINC01116. We observed that EGR1 was up-regulated in LAD cells compared with HBE (Fig. 2A). We noticed that pcDNA3.1/EGR1 could up-regulate EGR1 in A549 and PC9 cells (Fig. 2B). Subsequent qRT-PCR demonstrated an elevation in the expression of LINC01116 induced by overexpressing EGR1 (Fig. 2C), which indicated that EGR1 may stimulate the transcription of LINC01116. We then found the DNA motif of EGR1 and acquired three specific binding sites in the sequence of human LINC01116 promoter from JASPAR, as illustrated in Fig. 2D. We divided the promoter of LINC01116 into four parts according to the predicted binding sites (Fig. 2E). ChIP assay manifested that the LINC01116 promoter was remarkably pulled down by specific antibody targeting EGR1 only in P4 sectional part (Fig. 2F). To further testify this finding, we sub-cloned the full promoter region of LINC01116 (P FL) and P4-deleted promoter region into pGL3 vector (Fig. 2G). Luciferase reporter assay revealed that the luciferase activity of P FL was distinctively enhanced by overexpressing EGR1 in HEK-293 T, whereas no luciferase activity change in the deletion of P4 sectional part (Fig. 2H). This observation represented that EGR1 interacted with LINC01116 promoter at about − 1400 to − 2000 bp downstream the transcription start site (TSS). Since three binding sites were predicted by JASPAR in − 1400 to − 2000 bp of LINC01116 promoter, we point-mutated the sequence and constructed pGL3 luciferase reporter vectors containing P4-Mut1, P4-Mut2 and P4-Mut3 together with P4-WT, as shown in Fig. 2I. Luciferase reporter assay found that EGR1 overexpression promoted the activity of P4-WT, P4-Mut 2 and P4-Mut 3, while eliciting no effects on that of P4-Mut 1 whose sequence was mutated from − 1894 to − 1881 (Fig. 2J). Overall, LINC01116 was activated by transcription factor EGR1 at − 1894 to − 1881 sites in its promoter region.

### LINC01116 facilitates LAD oncogenicity by sponging miR-744-5p

**Fig. 3:**
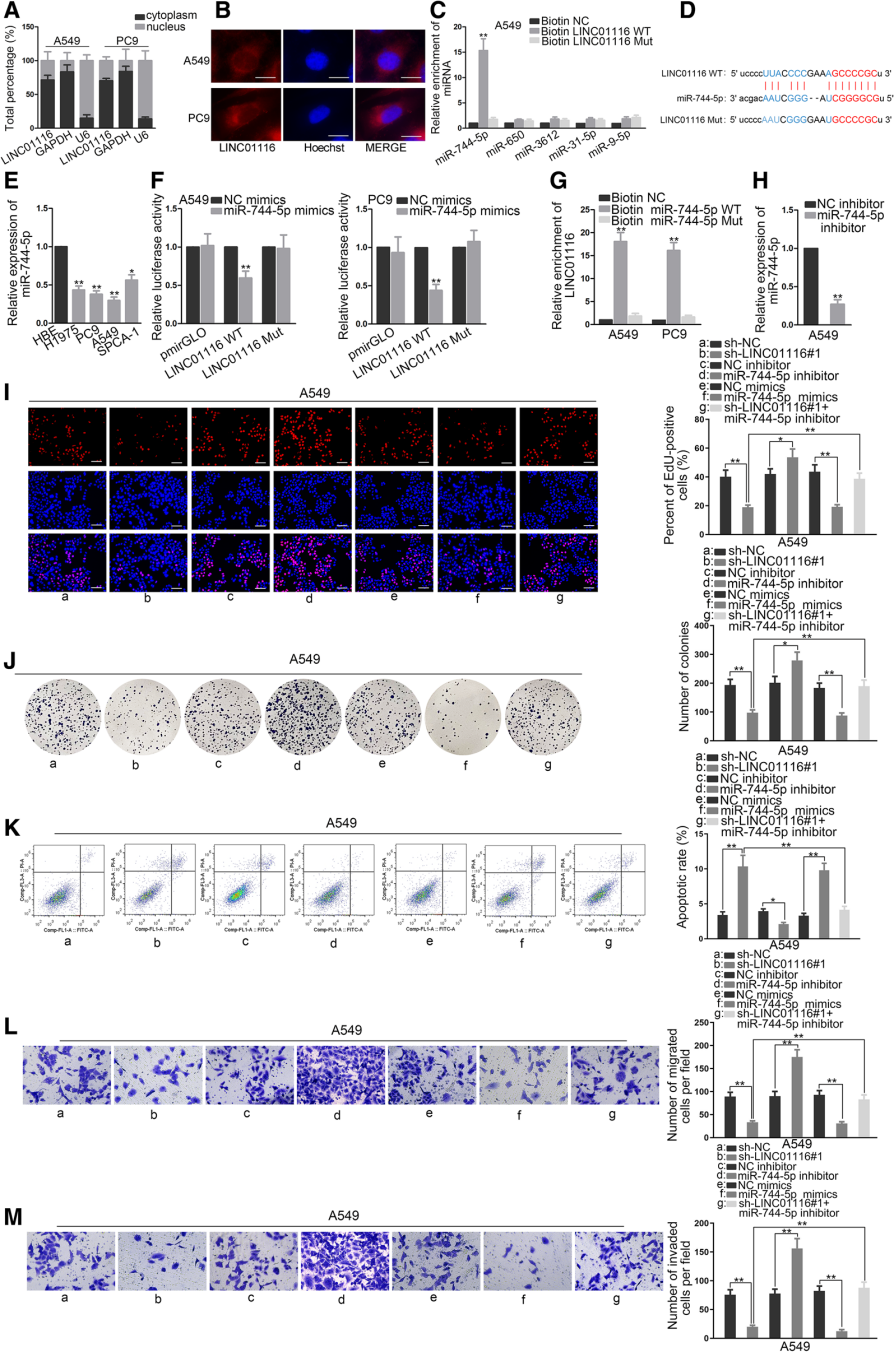
LINC01116 facilitates LAD oncogenicity by sponging miR-744-5p. **A**, **B** LINC01116 distribution in A549 and PC9 was explored by subcellular fractionation and FISH assays. Student’s t test was applied for analysis. **C** It was mirrored in RNA pull down assays that miR-744-5p was much enriched by Biotin LINC01116-WT. One-way ANOVA was applied for analysis. **D** Binding sites of miR-744-5p and LINC01116 were predicted. **E** Calculation of miR-744-5p expression in LAD cells and HBE was achieved by means of qRT-PCR. One-way ANOVA was applied for analysis. **F** LINC01116 fragment covering the wild-type or mutated miR-744-5p interacting sequences was inserted into pmirGLO luciferase vector. Luciferase activities were normalized to renilla luciferase. Student’s t test was applied for analysis. **G** RNA pull down verified the interaction between LINC01116 and miR-744-5p. One-way ANOVA was applied for analysis. **H** qRT-PCR analysis was applied for determining the efficiency of miR-744-5p inhibitor. Student’s t test was applied for analysis. **I**–**M** Function experiments and rescue assays were performed for detecting miR-744-5p influences on LAD and the rescuing effects of miR-744-5p inhibitor on suppressed cell malignant behaviors induced by LINC01116 knockdown. Student’s t test was applied for analysis between NC inhibitor and miR-744-5p inhibitor or between NC mimics and miR-744-5p mimics, while one-way ANOVA was applied for analysis among sh-NC, sh-LINC01116#1 and sh-LINC01116#1  +  miR-744-5p inhibitor. *P  <  0.05, **P  <  0.01

Previous study uncovered that cytoplasmic lncRNAs could regulate the progression of cancer via sponging certain miRNAs [19]. To determine whether LINC01116 could act as a sponge for miRNA, we explored the localization of LINC01116 in A549 and PC9 cells. Subcellular fraction and FISH assays revealed that LINC01116 was predominantly distributed in cytoplasm (Fig. 3A, B). Next, we utilized starBase (http://starbase.sysu.edu.cn/) bioinformatics analysis to find the potential miRNAs that could bind with LINC01116. Only miR-744-5p was found to be significantly enriched in biotin LINC01116-WT rather than biotin LINC01116-Mut (Fig. 3C). Later, qRT-PCR was carried out to evaluate the effect of LINC01116 knockdown on miR-744-5p in A549 and PC9 cells (Additional file 1: Figure S1C). The results indicated LINC01116 knockdown had no influence on miR-744-5p expression, showing that LINC01116 could competitively bind with miR-744-5p. In addition, starBase indicated putative binding sites between miR-744-5p and LINC01116 (Fig. 3D). Moreover, we found that the expression of miR-744-5p was markedly lower in LAD cell than that in HBE (Fig. 3E). Dual luciferase reporter assays suggested that the transfection of miR-744-5p mimics restrained the luciferase activity of LINC01116-WT, yet eliciting no variation in that of LINC01116-Mut (Fig. 3F). Additionally, RNA pull down assay manifested that LINC01116 was pulled down and enriched by Biotin miR-744-5p-WT probe (Fig. 3G). These data indicated that miR-744-5p could bind to LINC01116. Later, the function of miR-744-5p was investigated. We firstly silenced miR-744-5p utilizing miR-744-5p inhibitor (Fig. 3H). It was showed that miR-744-5p inhibitor obviously promoted A549 cell proliferation in EdU and colony formation, and counteracted the anti-proliferation effects of LINC01116 silencing (Fig. 3I, J). Furthermore, miR-744-5p inhibitor diminished apoptotic cells relative to control group, and reversed the pro-apoptosis influence exerted by LINC01116 silencing (Fig. 3K). Additionally, miR-744-5p inhibitor exerted positive impacts on migrating and invasive abilities of A549 cells, and offset the inhibitory impacts on migration and invasion induced by LINC01116 silencing (Fig. 3L, M). MiR-744-5p mimics elicited opposite outcomes in these cellular activities. Together, LINC01116 facilitated LAD oncogenicity by sponging miR-744-5p.

### LINC01116 mediates the expression of CDCA4 via competitively binding to miR-744-5p

**Fig. 4:**
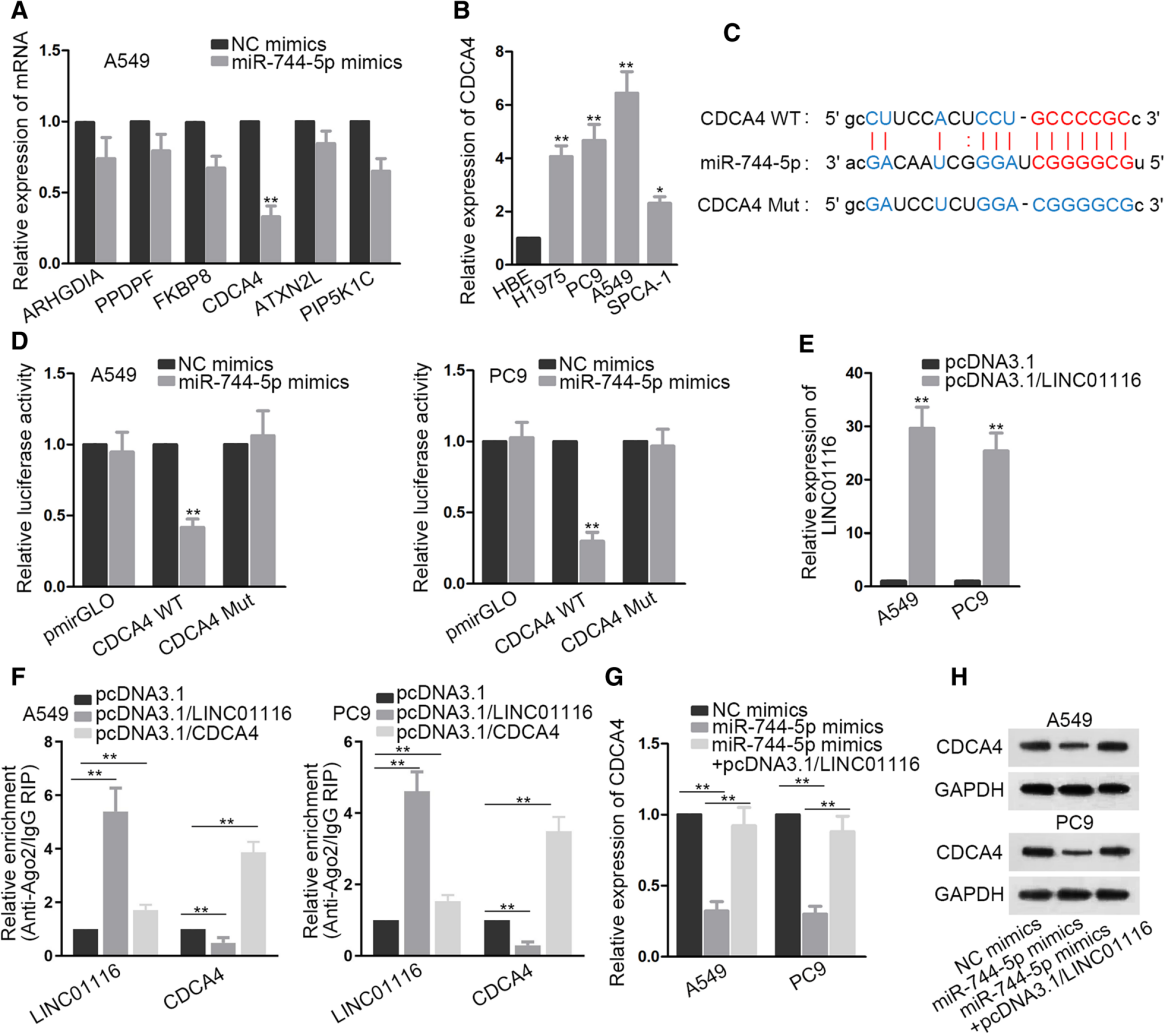
LINC01116 mediates CDCA4 expression via competitively binding to miR-744-5p. **A** qRT-PCR analysis was applied for quantifying expression levels of potentially combinable mRNAs of miR-744-5p in cells with miR-744-5p augment. Student’s t test was applied for analysis. **B** CDCA4 expression in LAD cell lines was determined by means of qRT-PCR. One-way ANOVA was applied for analysis. **C** Putative and mutant binding sites of miR-744-5p with CDCA4 were depicted. **D** It was reflected in Dual luciferase reporter assays that the decline in luciferase activity of CDCA4-WT resulted frommiR-744-5p mimics transfection. Student’s t test was applied for analysis. **E** The expression of LINC01116 was explored after transfection of pcDNA3.1/LINC01116 in A549 and PC9. Student’s t test was applied for analysis. **F** QRT-PCR based on RIP indicated that LINC01116 competed against CDCA4 for the binding with miR-744-5p. One-way ANOVA was applied for analysis. **G**, **H** The effects of miR-744-5p and LINC01116 overexpression on CDCA4 mRNA and protein levels were evaluated by qRT-PCR and western blot. One-way ANOVA was applied for analysis. *P  <  0.05, **P  <  0.01

Since miRNA was reported to affect the progression of cancers via suppressing downstream target gene expression and biological performance, we studied miR-744-5p mechanism in LAD via exploring potential downstream targets. Six candidate mRNAs that might bind to miR-744-5p were screened out by adopting starBase bioinformatics method (clip data: medium stringency  ≥  2; degratome data: medium stringency  ≥  2; program number: 2; AgoExpNum:  ≥  10). QRT-PCR analysis showed that CDCA4 was significantly down-regulated by transfection of miR-744-5p mimics into A549 cells (Fig. 4A). Subsequent qRT-PCR detected an abnormal overexpression pattern of CDCA4 in LAD cells (Fig. 4B). We then obtained putative binding sites between miR-744-5p and CDCA4 (Fig. 4C). Dual luciferase reporter assays showed that the luciferase activity of CDCA4-WT was notably decreased in A549 and PC9 by miR-744-5p overexpression (Fig. 4D). We transfected pcDNA3.1/LINC01116 into A549 and PC9 and found that the expression of LINC01116 significantly increased (Fig. 4E). Moreover, RIP assay was performed in A549 and PC9 cells with augmented LINC01116/CDCA4 levels. Then, qRT-PCR results based on RIP indicated that LINC01116 competed with CDCA4 for the binding to miR-744-5p (Fig. 4F). We also observed that CDCA4 was down-regulated as a result of transfection with miR-744-5p mimics, which was restored by co-transfection of pcDNA3.1/LINC01116 (Fig. 4G). Western blot showed the identical protein level variation, as shown in Fig. 4H. These observations were considered as additional evidence to the ceRNA role of LINC01116 in mediating CDCA4 expression via functioning as miR-744-5p sponge.

### LINC01116 facilitates LAD oncogenicity via targeting miR-744-5p/CDCA4 axis

**Fig. 5:**
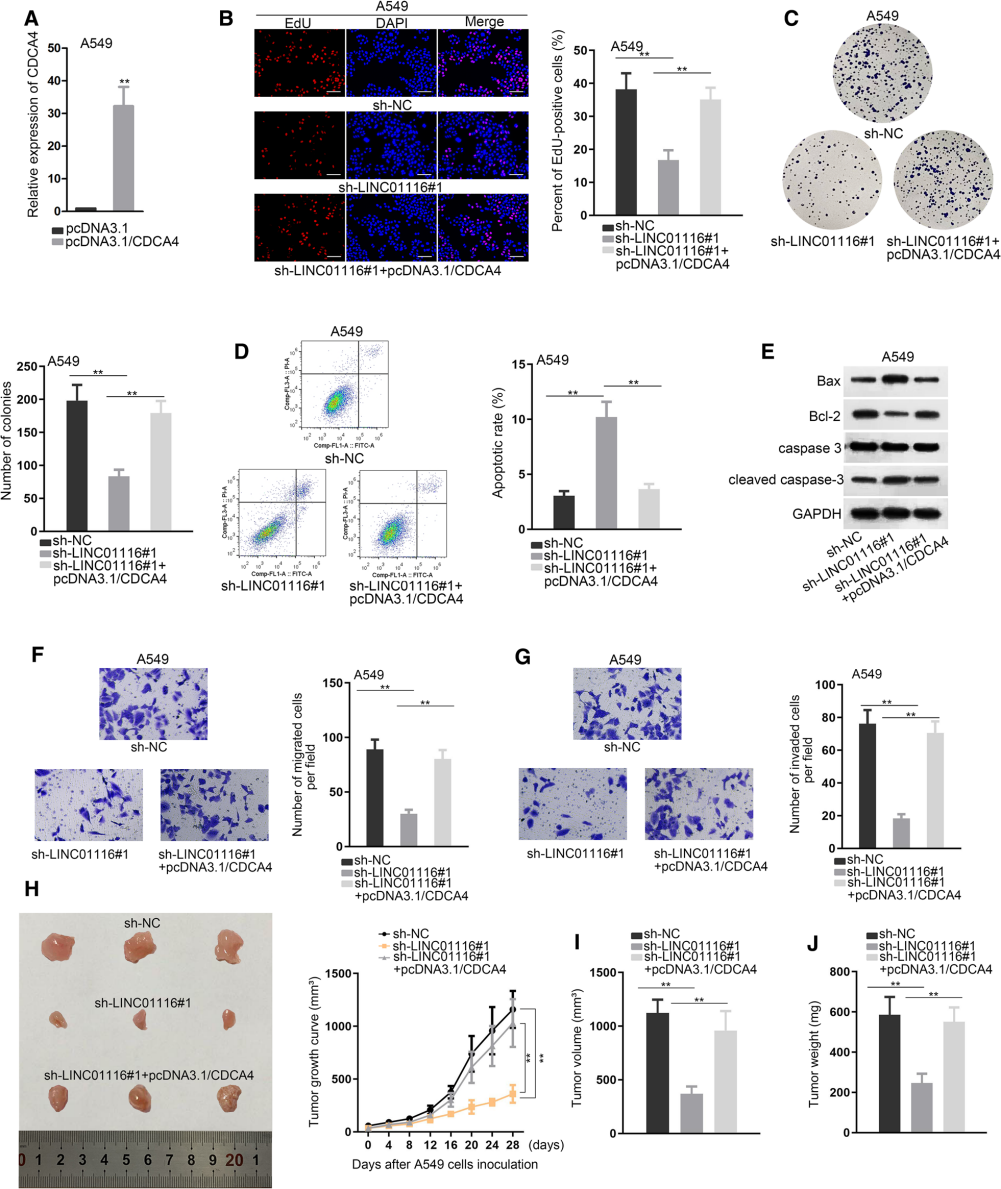
LINC01116 facilitates LAD oncogenicity via targeting miR-744-5p/CDCA4 axis. **A** The expression of CDCA4 was markedly up-regulated by pcDNA3.1/CDCA4. Student’s t test was applied for analysis. **B**, **C** EdU and colony formation assays were performed in A549 to study the effects of CDCA4 up-regulation on sh-LINC01116 induced cell proliferation. One-way ANOVA was applied for analysis. **D** Flow cytometry was conducted to evaluate cell apoptosis in a rescue analysis. One-way ANOVA was applied for analysis. **E** The variation of apoptosis-related proteins was investigated via western blot in a rescue analysis. **F**, **G** Transwell assays were performed for investigation into effects of CDCA4 up-regulation on sh-LINC01116 induced cell migration and invasion. One-way ANOVA was applied for analysis. **H** Xenograft tumor growth curve over 4 week was illustrated after A549 cells inoculation. One-way ANOVA was applied for analysis. **I**, **J** Xenograft tumor volume and weight were measured after inoculation of A549 cells transfected with different plasmids respectively. One-way ANOVA was applied for analysis. **P  <  0.01

After verification of the ceRNA role of LINC01116 in regulating miR-744-5p/CDCA4, we intended to further elucidate the effectiveness of LINC01116/miR-744-5p/CDCA4 axis in LAD. We used pcDNA3.1/CDCA4 to up-regulate the expression of CDCA4 in A549 cells (Fig. 5A). EdU assay showed that EdU positive cells were decreased by LINC01116 knockdown, but then recovered by CDCA4 up-regulation (Fig. 5B). Colony formation assays further proved such observation, with results showcased colonies were decreased by LINC01116 knockdown, but revived by CDCA4 up-regulation (Fig. 5C). In addition, strengthened cell apoptotic ability caused by LINC01116 knockdown was offset by CDCA4 up-regulation (Fig. 5D, E). Lastly, the attenuation on migration and invasion of A549 cells, resulting from LINC01116 knockdown, was counteracted by CDCA4 overexpression (Fig. 5F, G). All in all, CDCA4 overexpression reversed the inhibitory effects of LINC01116 silence on malignant behaviors of LAD cells. To further validate the inhibitory effects of LINC01116 silence on LAD oncogenicity, the xenograft model was established with inoculation of A549 cells steadily transfected with indicated plasmids. We found that silencing LINC01116 could hinder tumor growth, while this impact was offset by pcDNA3.1/CDCA4 (Fig. 5H). Consistently, significant decline in tumor volume and weight was discovered in response to LINC01116 depletion, but was reversed by pcDNA3.1/CDCA4 (Fig. 5I, J). Our findings uncovered that LINC01116 down-regulation restrained cell growth in vivo, and CDCA4 overexpression counteracted this effect of LINC01116 depletion on LAD oncogenicity.

## Discussion

Mounting researches have validated the crucial effects of aberrantly expressed lncRNAs on the course of multiple cancers, including LAD. LncRNA FBXL19-AS1 affected tumor growth and metastasis in LAD via acting as a miR-203a-3p sponge [20]. LncRNA TTN-AS1 promoted the progression of LAD via destabilizing PTEN protein, consequently activating PI3K/AKT signaling pathway [21]. These studies implied that research on cancer-associated lncRNAs may be conducive to learning the pathological molecular mechanism in LAD. LINC01116 was reported to play a carcinogenic role in glioma and may be a promising target for the clinical diagnosis and treatment of glioma [22]. Thus, we preliminarily assumed that it might possess oncogenic potential in LAD. LINC01116 was then found aberrantly overexpressed in LAD cells. In addition, LINC01116 knockdown could restrain proliferation, migration as well as invasion, yet facilitating apoptosis of LAD cells. In addition, we identified EGR1 as the transcription factor responsible for promoting the transcription of LINC01116.

LncRNAs mainly exert effects on cancers via participating in the ceRNA network through sponging special miRNAs to regulate relevant mRNA expression and protein output [23]. The role of lncRNA as a ceRNA in LAD has been revealed. LncRNA MNX1-AS1 promoted LAD progression through the regulation of miR-527/BRF2 axis [24]. Herein, we detected the abundance of LINC01116 in the cytoplasm of LAD cells. Mechanistically, LINC01116 was found to interact with miR-744-5p. MiR-744-5p was determined to repress cell proliferation in LAD via targeting MAFG [25]. In our study, miR-744-5p was targeted by LINC01116 in LAD and knockdown of miR-744-5p could promote the growth and migration of LAD cells. The discovery of miR-744-5p as a tumor suppressor in LAD was in line with the findings of previous studies.

A large quantity of studies has supported the regulating effect of miRNAs on target genes, which further affects the molecular biological function in various cancers [26–28]. The crucial regulator role of miRNAs in the development and advancement of LAD has been uncovered as well. For instance, miR‑505‑5p was found to suppress LAD cell apoptosis via targeting TP53AIP1, serving as a valuable diagnostic biomarker for early LAD diagnosis [25]. Moreover, miR-744-5p was reported to facilitate cell apoptosis by targeting HNRNPC and NFIX in ovarian cancer [15].In this study, CDCA4 was identified as the target gene of miR-744-5p and was negatively regulated by miR-744-5p. CDCA4 has been reported to significantly impact cell proliferation and apoptosis in human triple negative breast cancer [28]. Here, we firstly revealed that it had binding sites with miR-744-5p and could be restored by overexpressing LINC01116 in LAD cells. LINC01116 could restore the expression of CDCA4 via competing for the binding sites of miR-744-5p. Moreover, CDCA4 up-regulation markedly counteracted the inhibitory effects caused by silencing LINC01116 on malignant behaviors of LAD cells. At present, the functions of LINC01116 in other cancers have been discussed in various researches, while its function in LAD remains unclear. Therefore, the regulation of the LINC01116/miR-744-5p/CDCA4 axis in LAD needs confirmation in large-scale clinical studies, which could be a vital target in the future study.

## Conclusion

We discovered that LINC01116 contributed to cell proliferative, migrating and invasive capacities in LAD via sponging miR-744-5p to elevate the expression of CDCA4, providing some theoretical evidence of novel target for LAD treatment.

## Supplementary Information

**Additional file 1: Figure S1.** A. The levels of ki-67 and PCNA were detected via western blot to evaluate cell proliferation capability. B. The expression of LINC01116 was detected after silencing five transcription factor candidates. One-way ANOVA was applied for statistics analysis. C. Quantification of miR-744-5p was completed with the help of qRT-PCR after knockdown of LINC01116 in A549 and PC9 cells. Student’s t Test was applied to statistics analysis. **P  <  0.01.

## Data Availability

Research data are not shared.
