# Supplementary figures and images for: Long non-coding RNA LINC01116 is activated by EGR1 and facilitates lung adenocarcinoma oncogenicity via targeting miR-744-5p/CDCA4 axis
Source: Cancer Cell Int. 2021 Jun 5;21:292. doi: 10.1186/s12935-021-01994-w (PMC8180037; doi:10.1186/s12935-021-01994-w)

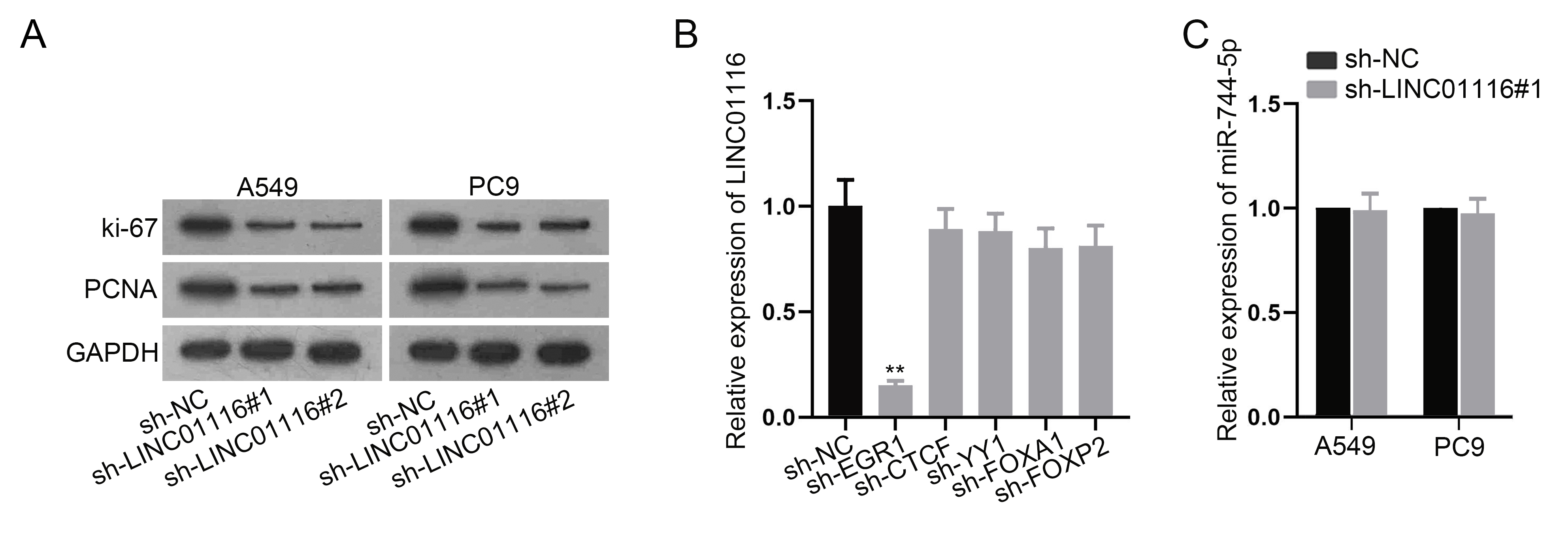

Supplement: Supplementary file 1 — Additional file 1: Figure S1. A. The levels of ki-67 and PCNA were detected via western blot to evaluate cell proliferation capability. B. The expression of LINC01116 was detected after silencing five transcription factor candidates. One-way ANOVA was applied for statistics analysis. C. Quantification of miR-744-5p was completed with the help of qRT-PCR after knockdown of LINC01116 in A549 and PC9 cells. Student’s t Test was applied to statistics analysis. **P < 0.01. [file 12935_2021_1994_MOESM1_ESM.tif]
